# Editorial

**Published:** 1854-10

**Authors:** 


### Editorial.

### THE DENTAL REGISTER.

The first number of the Eighth volume of this periodical is now before the Profession. It appears scarce possible that seven long years have passed away since we first assumed the labor, duty and responsibility of the editorial department. It appears as but yesterday, since the question was brought up in our Society, shall we have an organ for our Society and the Profession West? The subject was discussed with much anxiety, and notwithstanding the gloomy picture by an old veteran in the Medical Profession (Prof. D. Drake, M. D.,) whose experience was not very flattering, yet we have now entered our eighth year. Still we feel more and more of the onward and progressive spirit of Dental Science. Why not? Shall this bustling world move on with all its vast accelerated power, and we stand still? Nay! but the current bears us onward and we have no thought of standing still and let the car of Young America run over us.

For seven years we have tried to give the Profession everything new and useful which this progressive age has brought to light. Our periodical during this time has nearly doubled itself in size, and quite so in cost, yet we have not changed the price. Our aim has been and is now, to make it full as good as the patronage of the Profession will allow. Therefore, as our subscription list has gradually increased we have increased the size of the Register.

We commence the eighth volume with the determination to double this year the list of subscribers and hope the Profession will aid us in this effort. We shall therefore send the present number of the Register to several hundred who have heretofore not subscribed. And we would merely say to such, that if they do not wish to subscribe, they will please write their name and address on the envelope or cover of the Register and mail Dental Register, Cincinnati, Ohio. As we employ no traveling agents, this is the only way we can reach the

Profession. We hope, however, to make it such a work as the Profession, East as well as West, will be pleased to take.

In relation to the present number, we would say, that it has been gotten up under all the depressing influences of a long and oppressively hot Summer—rendering mental labor almost out of the question. This accounts for the scarcity of original matter. The heat and misquetoes have, we think, operated sadly on our contributors. It has given us, however, an opportunity of bringing up more fully our *Abstract* department which had fallen rather behind.

To our Eastern brethren we would say, that the Profession West possesses much of the spirit of Young America, and although not first in starting Dental Periodicals and Colleges, yet we are trying to do our duty in this way, and if not first in the latter, we claim to be first in the erection of a Temple exclusively for the benefit of Dental Science. That you may be better acquainted with the West, and know something of its unparalleled growth, and as we believe, future power and wealth, we shall send you the Register and hope that many who may not get it, will order it, in this way we shall make your acqaintance, hoping e'er long that the "iron horse" will bring us in close unison, when we shall welcome you in the Queen of Cities.

### OHIO COLLEGE OF DENTAL SURGERY.

We would say to the stockholders at a distance, and the Profession generally, that the new building in process of erection this summer, for this Institution, is now completed and fully equals our expectations in every particular. We have two as neat and commodious Lecture rooms as can be found in the city—each large enough to seat from 125 to 150 students. We have three dissecting rooms in all their arrangements admirably suited for the purpose designed. Our Laboratory is large and commodious, and we would say to students that our Infirmary rooms, being four in number, are especially arranged with reference to the details of office practice. Such rules and regulations as should be observed in private practice will here be carried out, thus giving the student an induction into the proprieties of professional life. The students will be arranged in classes and only one class, of perhaps two, allowed in one room at a time unless in *important operations.* In this way the patient's feelings will be respected and the student under less restraint. The Library and Museum rooms are well adapted for the purpose. The building indeed throughout is well adapted for the purpose of Dental Instruction. It is substantially built, and we hope may stand for at least one century as a memorial of the Professional zeal of the *Profession* in this great valley.

The regular course of lectures will commence in this Institution, the first Monday of November and continue between sixteen and seventeen weeks.

### AMERICAN SOCIETY OF DENTAL SURGEONS.

We are as yet uninformed when the meeting of this Society will take place Due notice will of course be given.

### DENTAL RECORDER.

The last number of this excellent periodical contains the valedictory of Dr. Hill, who retires from the Editorial chair. It is with regret we part with the Doctor, and hope that it may not be the means of his laying aside also his pen. We think that those who can write and are some in the habit of it, should feel it a duty to labor in this way. Our profession needs continued labor in this particular, to give it that standing it is now making rapid strides to attain.

We are sorry to hear that the Doctor's labor in this way has not proved more profitable. The "experience is, however, worth something," and we hope this will amply repay.

Messrs. Sutton and Raynors, for the present, take charge of the Recorder, expecting soon to fill Dr. Hill's place with a gentlemen every way qualified to discharge its duties. We still say to the Recorder, go on, and wish it much success. Will Dr. Hill please send us the two last (July and August) numbers of the Recorder. If they ever came to hand they have been mislaid and we had the other day to borrow them.

### DENTAL SUPPLIES.

We have once before spoken of the increased facilities afforded the Profession in the way of Dental Supplies. It appears, indeed, that however rapid the advance of the Profession in all its different departments, that this very necessary and important branch progresses in an equal ratio.

The manufacturing department embracing the manufacture of Teeth, Instruments, Gold and other necessaries for successful dental practice, is certainly making great advancement, and we are especially reminded of this by looking over the late stock of teeth and materials laid in by Dr. J. M. Brown, who has just returned from the East.

The Doctor appears determined to supply the Profession with everything needful and new. To his already extensive stock, he has recently added a fine lot of Messrs. Orum & Armstrong's beautiful Teeth. It appears that these gentlemen are determined to rival the well known establishment of Jones, White, and McCurdy. They are taking great pains in the shaping and coloring of their teeth and have secured a very fine and life-like gum. If their teeth prove to be as strong as Jones, White and McCurdy's, they will certainly come in strong competition.

Dr. Brown is agent for both of these establishments, and the Profession have reason to thank the Dr. for his breaking in on the old practice of exclusive agencies. We have always said, bring these things into competition and let the most meritorious take the lead. Dr. Brown's assortment is now large and well selected.

### OBITUARY.

Dr. Ludolph Parmly, of Mobile, Alabama, we are sorry to see, deceased in July last. Dr. Parmly has enjoyed a high reputation and justly may be regarded as one of the ornaments of our profession.

### NELSON AMERICAN LANCET.

We have on our table the September number of this valuable monthly. It is ably edited by Drs. Horace and Alfred Nelson, printed and published at the Lancet office, Montreal, Canada East, and Plattsburg, New York. The style of the work and typographical execution is fine. The work is well filled with original matter.

### RESIGNATION.

We see by the last number of the Journal, that Professor Townsend of the Philadelphia College of Dental Surgery, has resigned his chair, which he has filled with much credit to himself. We regret to hear that ill health is the cause. The chair, we understand will soon be filled by a very competent individual.

### GOLD FOIL.

We refer our readers to the card of R. H. Ransley—King's Manufactory, had a long and well deserved reputation, and we believe its character has been well sustained by Mr. Ransley. We have used some of the latter's manufacture, which we like very much. The Profession will find their orders filled with promptness and fidelity.

### DENTAL PATENTS.

The article on Dental Patents, which we have given place to, in the present number of the Register, is by mistake of the printer, curtailed some two pages. This we will give in our next number so as to present the Doctor's argument in full.

### E. E. JOHNSON'S SUPERIOR TOOTH SOAP.

We would inform Mr. Johnson that the box of Soap has just come to hand, but too late to attend to his request in full. We like it far better than any Soap we have ever used, and hope he will supply Dr. Brown, his agent, with it. We would like the Profession to try it.

